# A randomized controlled trial investigating the effect of thermal steam spray on nasal condition, neurological function, and sleep quality in young adults with nasal congestion and irregular sleep patterns

**DOI:** 10.1186/s12890-026-04585-5

**Published:** 2026-08-13

**Authors:** Jaehoon Kim, Jaehoon Hwang, Kyungryul Chung, Youngsoo Jin, Yong-Seok Jee

**Affiliations:** 1 Korea Sleep Industry Association, Gangnam-daero 34, Seocho-gu, Seoul, 06738 Korea; 2 Korea, Republic of South Korea; 3 Department of Public Health Special Education, Graduate School of Health Promotion, Hanseo University, #1 Hanseo-ro, Haemi-myeon, Seosan, 31962 Korea; 4 Rhinocare Korea Inc, Sanho-daero 253, Gumi-si, Gyeongsangbuk-do 39371 Korea

**Keywords:** Intranasal steam, Nasal congestion, Sleep quality, Autonomic nervous system, Electroencephalography, Heart rate variability, Respiratory function

## Abstract

**Background:**

Intranasal thermal steam spray (ITSS) has been suggested to relieve nasal stuffiness and improve sleep-related symptoms. However, its mechanistic effects on respiratory capacity, neurological functions, including autonomic nervous system (ANS) and brain activities, remain underexplored. Therefore, this study aimed to investigate the effects of an ITSS intervention on nasal condition, respiratory and neurological functions, and sleep quality in young adults with nasal congestion and irregular sleep patterns.

**Methods:**

Sixty university students (aged 20–26 years) with an average duration of nasal congestion and sleep irregularity of over three years participated. Two dormitories were randomly assigned to either a control group (CON; *n* = 30) or an intervention group (ITSS; *n* = 30). The ITSS device, delivering 43 °C thermal wet steam using jet stream (80 mm H₂O) technology, was administered for 15 min, 3 times per week, 30 min before bedtime for 8 weeks.

**Results:**

After the intervention, subjective nasal congestion and sleep conditions deteriorated in the CON but improved in the ITSS (*p* < 0.001). Pulmonary outcomes, including reduced respiratory rate (*p* < 0.001) and increased FEV₁ (*p* < 0.001) and PEF (*p* < 0.001), improved only in the ITSS. Heart rate variability and blood pressure indices also showed favourable changes. Electroencephalographic analysis revealed increased wide band, alpha, and gamma powers in the ITSS, contrasting with declines in the CON (*p* < 0.05). In the ITSS group, PSQI-K components improved, with daytime dysfunction reduced by 32.78 ± 41.40% (*p* < 0.001). Total PSQI-K scores rose in the CON group (+ 14.64 ± 22.44%) but declined in the ITSS group (− 38.90 ± 21.99%), showing a significant difference (*p* < 0.001).

**Conclusions:**

These findings suggest that ITSS enhances airflow by humidifying the nasal environment, thereby facilitating respiratory function and stabilizing ANS activity and brain function. This process ultimately contributes to improved overall sleep quality in young adults experiencing nasal congestion and irregular sleep patterns.

**Trial registration:**

The study was retrospectively registered on April 3, 2025, in the Clinical Research Information Service (https://cris.nih.go.kr/cris/search/listDetail.do/) under registration number KCT0010381.

**Supplementary Information:**

The online version contains supplementary material available at https://doi.org/10.1186/s12890-026-04585-5.

## Background

Respiration is an essential life-sustaining activity that delivers oxygen to all cells of the human body, enabling normal physiological functions. While breathing can occur through both the nasal and oral cavities, the nose is the primary organ responsible for respiratory capacity [1]. The respiratory system comprises from nasal cavity to lungs, facilitating oxygen intake through the nasal passages and its circulation throughout the body via alveoli in the lungs [2]. For efficient respiration, the intranasal airway must remain unobstructed [3]. However, various conditions such as nasal septal deviation, colds, sinusitis, and allergic rhinitis can lead to nasal congestion or obstruction [4]. Nasal congestion including abnormal rhinitis or can compromise nasal respiratory function, thereby disrupting the sleep–wake cycle [5]. Such impairment has been associated with various sleep disorders, including insomnia, hypersomnia, and so on [6]. Irregular sleep patterns—especially those linked to nasal dysfunction—have been shown to impair respiratory function and are further associated with elevated stress levels, increased fatigue, and declines even in cognitive performance and concentration [7]. Especially, nasal obstruction may influence autonomic nervous system (ANS) regulation by increasing upper airway resistance and altering breathing patterns [8]. Increased respiratory effort and impaired nasal airflow have been associated with sympathetic activation and reduced parasympathetic modulation, resulting in alterations in heart rate variability (HRV) and cardiovascular regulation [9]. Consequently, respiratory function, blood pressure (BP), and HRV represent complementary physiological indicators that may reflect changes in autonomic regulation associated with improvements in the nasal environment [10]. Ultimately, maintaining an intranasal airway is a crucial factor in ensuring a high quality of life, sleep quality, and overall health [11, 12].

Previous studies have investigated conservative approaches for improving the nasal environment, including nasal irrigation and conventional steam inhalation [13–15]. Although these methods are safe and inexpensive, conventional steam inhalation often requires preparation of heated water or external steam-generating equipment and may provide inconsistent steam temperature and exposure conditions [16]. In contrast, the investigational intranasal thermal steam spray device (ITSS) used in the present study is a portable, temperature-controlled system designed to deliver warm humidified steam directly into the nasal cavity under standardized operating conditions. The present study was therefore designed to evaluate whether these practical design characteristics are associated with improvements in nasal condition, respiratory function, neurological function, and sleep quality. This home medical device has been reported to provide a safe and effective solution for nasal congestion by delivering moist steam at 43 °C into the nasal cavity [17]. However, despite its demonstrated effectiveness in alleviating intranasal congestion, no studies to date have explored its potential impact on respiratory capacity and sleep quality. In particular, there is a significant lack of research investigating whether the flow of air circulating through an optimal respiratory pathway influences the ANS regulation and, in turn, affects stable brain activity. Therefore, the primary objective of the present randomized controlled trial was to determine whether improving the nasal environment through ITSS could enhance sleep quality in young adults with nasal congestion and irregular sleep patterns. A secondary objective was to investigate potential physiological mechanisms underlying these effects by evaluating respiratory function, ANS activity, and electroencephalographic responses.

## Materials and methods

### Study design

This study was designed as a randomized, double-blinded, sham-controlled trial. Following approval by the Hanseo University Institutional Review Board (HS24-12-18), the study investigated the efficacy of ITSS through an interventional approach. Participants underwent an 8-week ITSS intervention (April 7 to June 2, 2025), followed by assessments and analyses of its impact on subjective nasal congestion, perceived sleep condition, respiratory performance, neurological function including ANS and brain activities, and overall sleep quality. To preserve participant blinding, the active and sham devices were identical in external appearance, weight, indicator lights, operating sound, and handling procedures. They were informed only that two different versions of the investigational device were being compared and were not informed which device represented the active intervention. All intervention sessions were conducted individually, and participants were instructed not to discuss their experiences with other participants throughout the study period. Outcome assessors and data analysts remained blinded to group allocation until completion of the statistical analyses. This study obtained written informed consent in accordance with the Declaration of Helsinki [18] and was retrospectively registered with the Clinical Research Information Service, Republic of Korea (registration number: KCT0010381) on April 3, 2025. The trial was registered retrospectively because of an administrative oversight in the timing of registration. Importantly, the study protocol, eligibility criteria, intervention procedures, outcome measures, and statistical analysis plan had been finalized before participant enrolment, and no protocol modifications were made as a result of the delayed registration. This study adhered to the CONSORT guidelines published in 2025 [19], and the completed checklist is provided in the supplementary materials (Supplementary file 1).

### Participants

The participants were male and female university students aged between 20 and 26 years, residing in two dormitories on the same university campus. They expressed interest in the study after viewing a social media advertisement related to the research, reporting symptoms of nasal congestion and sleep disturbances. Participants were instructed to carry out their daily activities and meals within their assigned dormitories and were asked not to disclose their involvement in the study to others. The inclusion criteria for this study required participants to exhibit symptoms such as clear nasal discharge, sneezing, nasal congestion, and nasal itching, along with irregular sleep patterns. Participants were assessed to determine whether their symptoms were occasional or persistent and to identify periods of symptom exacerbation. Subsequently, an otolaryngologist conducted a diagnostic evaluation for rhinitis, including nasal examination and nasal secretion analysis. Meanwhile, individuals diagnosed with congenital nasal obstruction, such as congenital pyriform aperture stenosis, or those with chronic diseases affecting the heart, lungs, or other major organs were excluded from this study. Additionally, individuals who had been regularly participating in an exercise program for more than six months, were heavy smokers, engaged in high-risk alcohol consumption as defined by the WHO, or had metabolic syndrome were also excluded [20]. Individuals with a prior diagnosis of cardiovascular or cerebrovascular disease, cancer, or psychiatric conditions were likewise excluded from the study. The sample size was estimated using G*Power version 3.1.9.7 (Heinrich-Heine-Universität, Düsseldorf, Germany) for a repeated-measures design involving two groups and two measurement occasions [21]. Assuming a medium group-by-time interaction effect, an α level of 0.05, and a statistical power of 0.95, the minimum required sample size was estimated to be 54 participants. This calculation was based on the overall repeated-measures design rather than on an outcome-specific effect estimate. Therefore, the study was not necessarily powered to detect effects across all secondary and mechanistic outcomes, and these findings should be interpreted accordingly.

**Fig. 1:**
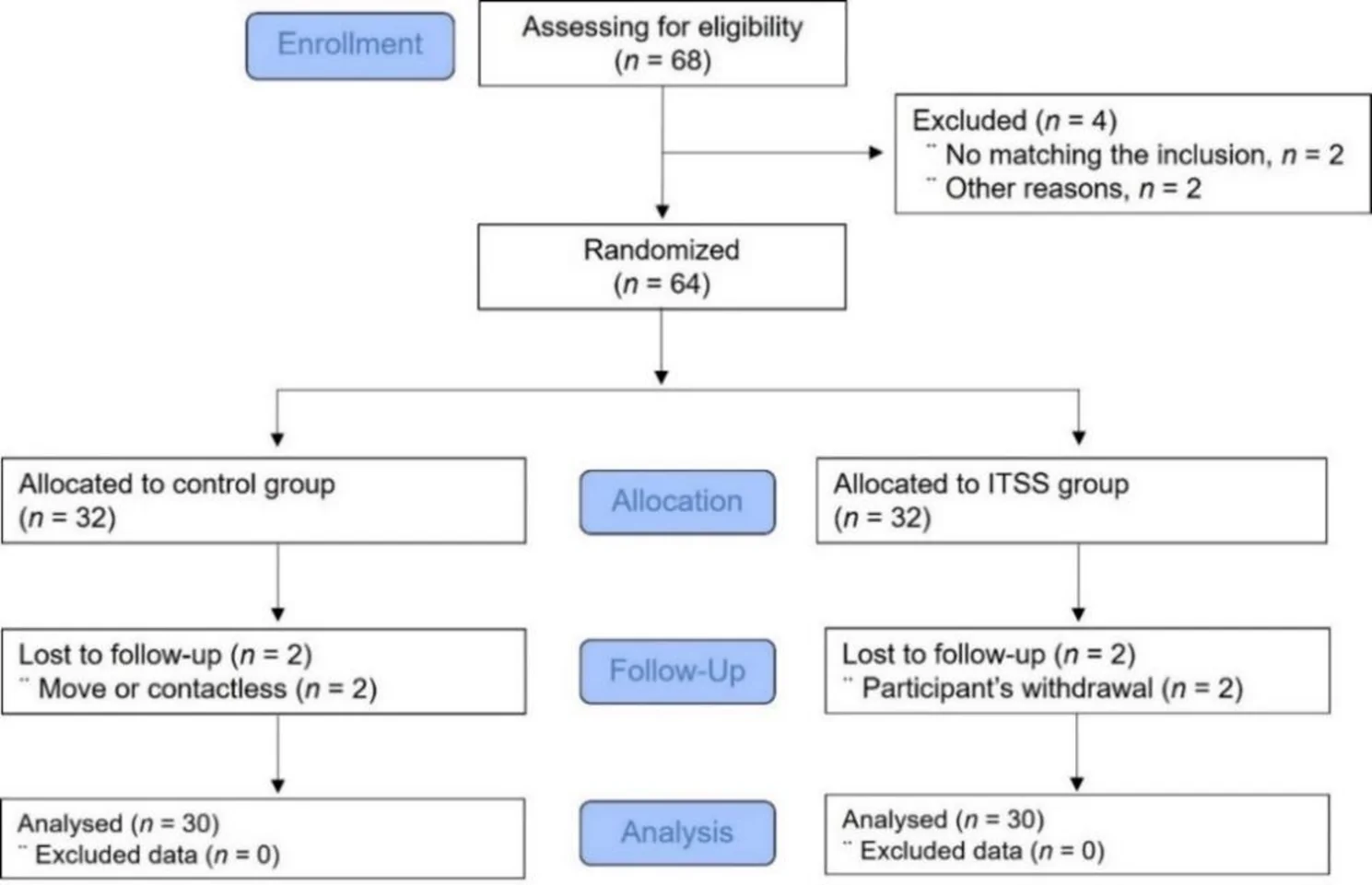
CONSORT flow diagram

As shown in Fig. 1, a total of 68 participants were recruited; however, 4 were excluded because they failed to meet the inclusion criteria or for personal reasons. Among the remaining 64 participants who completed the demographic survey and baseline assessment, random tags were used to assign one facility to the control group (CON) and another facility to the ITSS group. The principal investigator carried out all these procedures. A total of 32 participants were assigned to the CON at one facility, while another 32 participants were allocated to the ITSS group at a separate facility. During the eight-week program, two participants from the CON and two participants from the ITSS group dropped out during the follow-up stage. No participants from either group were excluded during the analysis, resulting in a final sample of 30 participants in each group: the CON and the ITSS group. Standardized guidelines were implemented for pre- and post-assessments to ensure strict blinding throughout the study.

### ITSS intervention program

An intranasal vapor spray device (RhinoCare Korea Inc., Gumi, Korea) is a medical device certified by the Ministry of Food and Drug Safety for the treatment of nasal congestion caused by rhinitis, sinusitis, colds, and flu. This device facilitates breathing by utilizing Jet Stream technology (80 mmH₂O), which delivers 43 °C vapor heat to the intranasal cavity through an internal heating mechanism, and its effectiveness has been recognized. Additionally, it has met safety standards for medical devices, including the residual wave test, as verified by an authorized testing agency [17]. Participants in the ITSS group were instructed to use the device for 15 min, three times per week, for eight weeks, activating it 30 min before bedtime. Meanwhile, participants in the CON were provided with the device without vapor heat emission and were instructed to use it under the same conditions as the ITSS group.

### Outcome measures

The primary outcome was sleep quality, evaluated using the Pittsburgh Sleep Quality Index–Korean version (PSQI-K). Secondary outcomes included subjective nasal congestion, respiratory function, BP, ANS activity assessed by HRV, and electroencephalographic parameters. These secondary outcomes were selected to explore potential physiological mechanisms linking improvement of the nasal environment to changes in sleep quality. Meanwhile, body composition and dietary intake were assessed to characterize the study population and to monitor potential confounding factors throughout the intervention period. Because nutritional intake, body composition, and obesity-related characteristics may influence respiratory function, nasal symptoms, and sleep quality, these variables were monitored to confirm baseline comparability and to minimize the potential influence of non-intervention-related factors on the study outcomes.

### Anthropometric data

These measurements were conducted on the first and final days of the intervention between 7:00 a.m. and 8:00 a.m., prior to breakfast. To ensure measurement accuracy, assessments were performed on participants who had refrained from fluid intake and had completed both urination and defecation beforehand. Height was measured using an automated stadiometer. Body weight, fat mass, and muscle mass were assessed using the InBody 720 device (BioSpace, Seoul, Korea) with participants standing after a 10-hour fasting period. Body mass index (BMI) was also calculated based on the weight-to-height ratio.

### Clinical data

To monitor external variables, participants were asked to complete a questionnaire between 9:00 p.m. and 10:00 p.m. each night prior to sleep, recording dietary intake and daily activities. In addition, between 7:00 a.m. and 8:00 a.m. each morning, the degree of subjective nasal openness and perceived post-sleep condition were assessed using a visual analogue scale (VAS). First, all participants in both groups consumed three meals per day, prepared under the supervision of a certified nutritionist. To ensure accurate dietary assessment and monitor adherence throughout the intervention, participants’ daily food intake was documented by a researcher using an AI-assisted dietary camera system (DoingLab Inc., Seoul, Korea) [22]. (Supplementary file 2). Second, physical activity (PA) levels were assessed using the International PA Questionnaire-Short Form [23] (Supplementary file 3). Third, all participants were instructed to evaluate the subjectively felt degree of nasal congestion and their post-sleep condition each morning upon waking, using a VAS-based recording sheet delivered via direct message. The perceived degree of nasal dilatation was determined using a VAS [24]. The left end of a 10 cm long line was placed as ‘open nasal cavity (0)’ and the right end was placed as ‘blocked nasal cavity (10)’, and they were asked to mark the degree of nasal congestion they felt. The left end of a 10 cm long line was placed as ‘no discomfort with sleep (0)’ and ‘severe discomfort with sleep (10)’, and they were asked to mark the sleep problems he or she felt. The collected data were analysed on a weekly and monthly basis.

### Respiratory capacity, blood pressure and heart rate variability

Respiratory capacity, BP, and HRV used to assess ANS function was measured between 7:00 a.m. and 8:00 a.m. on the first and final days of the intervention. First, resting respiratory rate was assessed by observing chest movement while participants lay in a supine position on a bed for 60 s. Subsequently, pulmonary function was evaluated using a Spirovit SP-1 spirometer (SCHILLER AG, Switzerland). Key respiratory variables measured included forced expiratory volume in one second (FEV₁) and peak expiratory flow (PEF). For testing, participants wore a nasal clip to prevent air leakage and inserted a disposable mouthpiece into their mouths. They were instructed to exhale forcefully through the mouth following a maximal inhalation to measure the targeted respiratory parameters. Prior to the actual data collection, participants underwent practice sessions to familiarize themselves with the spirometry technique. Each participant performed the test three times, and the highest value recorded among the trials was used for statistical analysis [25]. Second, resting BP was measured using an automated sphygmomanometer. For the assessment, an appropriately sized cuff was placed on the brachial artery of the right arm. Third, to assess ANS activity, HRV was measured using the uBiomacpa device (Biosensecreative Co. Ltd. Seoul, Korea). The measurements were taken twice on the day of the experiment, and the average values were used for analysis [26–29]. During the measurement, participants sat comfortably for 2–3 min while pulse waves were non-invasively recorded via a sensor attached to the index finger. To ensure data reliability, participants were advised to remain still and avoid talking, coughing, or deep breathing during the procedure. The analysis included both time-domain measures and frequency-domain parameters derived from power spectral density estimates using autoregressive modeling [30]. In time-domain analysis, HRV is assessed by measuring the intervals between successive normal QRS complexes, referred to as normal-to-normal (NN) intervals. Two key time-domain metrics are used: the standard deviation of NN intervals (SDNN), which reflects overall HRV by capturing all cyclic components, and the root mean square of successive differences between NN intervals (RMSSD), which primarily reflects parasympathetic activity. In contrast, frequency-domain analysis examines the spectral components of HRV. This includes low-frequency (LF) and high-frequency (HF) bands, which are associated with sympathetic and parasympathetic nervous system activity, respectively. The LF/HF ratio, calculated from normalized LF and HF values, served as an index of sympathetic modulation [31]. To enhance data accuracy, the first hour of recording was excluded from analysis [26], and any transient noise or movement artifacts were manually removed.

### Electroencephalogram data measures

Prior to electroencephalography (EEG) acquisition, participants underwent a 10-min resting period in a quiet and thermally controlled indoor environment to ensure physiological stabilization. Subsequently, resting EEG was recorded for 5 min while participants remained seated in a relaxed posture with their eyes gently closed [32, 33]. EEG data were acquired using a 10-channel wired EEG system (Neurofeedback Intermediate System, Montreal, Quebec, Canada). Scalp electrodes were affixed according to the international 10–20 system, targeting the frontal (F3, F4, Fz), temporal (T3, T4), parietal (P3, P4), and central regions. Reference electrodes were attached via ear clips on both earlobes. The sampling rate was configured at 500 Hz. Signal preprocessing included the application of a 60 Hz notch filter to remove power line interference and a bandpass filter to attenuate low- and high-frequency noise. Ocular artifacts were eliminated using Independent Component Analysis. Power spectral analysis was subsequently performed using Fast Fourier Transform, yielding absolute and relative power values across predefined frequency bands. To investigate the potential effects of nasal congestion on alterations in sleep-related EEG patterns, spectral power was examined across the entire brain in the wide band range and within specific frequency domains: delta (0.5–4 Hz; relative power: 0–20%), theta (4–8 Hz; 5–20%), alpha (8–12 Hz; 20–40%), beta (13–30 Hz; 10–30%), and gamma (> 30 Hz; 3–10%) [34]. Furthermore, to enable a more refined assessment of neural activity, the alpha / beta ratio (ABR) and theta / beta ratio (TBR) were computed and analyzed.

### Sleep quality measures

The Pittsburgh Sleep Quality Index (PSQI) is a standardized self-report questionnaire designed to assess sleep quality and patterns over the past month. It is widely utilized in clinical and research settings to evaluate sleep disturbances and insomnia. Originally developed by Buysse et al. [35], the PSQI has been adapted into various languages, including Korean. This study employed the Korean version of the PSQI (PSQI-K), which comprises 18 items across seven domains: subjective sleep quality (1 item), sleep latency (2 items), sleep duration (1 item), sleep efficiency (2 items), sleep disturbances (9 items), use of sleep medication (1 item), and daytime dysfunction (2 items). The PSQI-K has demonstrated high reliability, with a Cronbach’s α of 0.779. Each domain is scored on a scale of 0 to 3, yielding a total score ranging from 0 to 21, where higher scores indicate poorer sleep quality. In this study, sleep quality was classified based on the criteria established by Shon et al. [36]. A PSQI-K total score of 8.5 points or lower was categorized as “good sleep quality,” while a score exceeding 8.5 points was classified as “poor sleep quality.”

### Statistical analysis

Statistical analyses were performed using GraphPad Prism 10.4.1 software (GraphPad Software, Boston, MA, USA). Descriptive statistics, including the mean and standard deviation (SD) of all data, were calculated. The normality of variable distribution was assessed using the Kolmogorov-Smirnov test. Categorical variables were expressed as frequencies and percentages and analysed using appropriate statistical tests. To examine baseline differences prior to the main analysis, the Mann–Whitney U test was employed for non-parametric pre-intervention variables. For normally distributed data, a two-way repeated measures ANOVA (2 × 2) was conducted to assess group and time interactions. In cases of non-normally distributed data, between-group comparisons were performed using the Mann–Whitney U test, while within-group comparisons were assessed using the Wilcoxon signed-rank test. Because each outcome variable represented a prespecified physiological or clinical endpoint, statistical analyses were performed separately for each outcome. The primary interpretation focused on the group × time interaction rather than multiple pairwise comparisons. Percentage change (Δ%) was calculated to evaluate detailed variations at each time point. Effect sizes (η²) for the two groups were interpreted based on the thresholds proposed by Fritz et al. [37], with values < 0.10 considered small, 0.42 as medium, and 0.80 as large. The statistical significance level was set at *p* ≤ 0.05.

## Results

### Demographic and clinical factors’ characteristics

A total of 60 participants were randomly assigned to either the sham CON (males, *n* = 15; females, *n* = 15) or the ITSS group (males, *n* = 16; females, *n* = 14). The mean age of all participants was 22.27 ± 1.80 years, with an average height of 165.16 ± 9.03 cm, body weight of 68.82 ± 13.94 kg, and BMI of 24.98 ± 3.11 kg/m². All participants had the average durations of nasal congestion and irregular sleep pattern of 3.39 ± 0.74 years and 3.01 ± 0.77 years. Prior to the experiment, there were no significant differences between the groups in terms of age, sex, height, body composition, smoking status, and alcohol consumption. No significant differences were found between the groups regarding the durations of nasal congestion and irregular sleep duration. The pre-experiment characteristics of the two groups are presented in Table 1.

**Table 1:** Differences in demographic and clinical characteristics at baseline

	Groups						
	CON		ITSS				
Items	mean	SD	mean	SD	Z	*p*	η²
Age (y)	22.03	1.79	22.50	1.81	-1.023	0.307	0.017
Sex	1.50	0.51	1.47	0.51	-0.256	0.798	0.001
Height (cm)	163.91	9.39	166.41	8.63	-1.089	0.276	0.020
Weight (kg)	68.10	14.42	69.53	13.65	-0.459	0.646	0.003
Muscle mass (kg)	29.12	6.65	28.74	6.74	-0.067	0.947	0.001
Fat mass (kg)	19.98	5.00	20.50	5.99	-0.319	0.750	0.002
Percent fat (%)	30.61	10.09	30.47	10.23	-0.237	0.813	0.000
Body mass index (kg/m²)	25.09	3.31	24.88	2.95	-0.200	0.842	0.001
Basal metabolic rate (kcal)	1454.27	272.35	1466.00	251.51	-0.438	0.661	0.001
Smoking	1.47	0.51	1.43	0.50	-0.257	0.797	0.001
Alcohol	1.73	0.45	1.80	0.41	-0.605	0.545	0.006
Nasal congestion (y)	3.33	0.80	3.44	0.68	-1.093	0.275	0.006
Sleep irregularity (y)	2.98	0.73	3.03	0.82	-0.074	0.941	0.001

All data are presented as mean and standard deviation (SD). Sex was coded as male = 1 and female = 2, smoking status as smoker = 1 and non-smoker = 2, and alcohol consumption as drinker = 1 and non-drinker = 2 for analysis. *CON* control, *ITSS* intranasal thermal steam spray

Among the controlled variables, prior to the experiment, the dietary intake was 2251.73 ± 149.87 kcal for the CON and 2221.20 ± 164.69 kcal for the ITSS group, with no significant difference between the two groups (*p* = 0.630, η² = 0.010). After the experiment, the dietary intake measured 2380.80 ± 281.42 kcal in the CON and 2350.33 ± 219.76 kcal in the ITSS group, again showing no significant difference (*p* = 0.589, η² = 0.004). Similarly, before the experiment, the PA levels were 1814.53 ± 335.39 MET·min/ week for the CON and 1917.60 ± 360.07 MET·min/week for the ITSS group, with no significant difference (*p* = 0.294, η² = 0.022). After the experiment, the PA levels were recorded as 1730.40 ± 289.26 MET·min/week in the CON and 1750.43 ± 274.50 MET·min/week in the ITSS group, also showing no significant difference (*p* = 0.620, η² = 0.001). Meanwhile, throughout the 8-week intervention period, participants were monitored for potential adverse events, including nasal irritation, discomfort, excessive heat sensation, epistaxis, dizziness, and other unexpected symptoms following device use. No serious adverse events, treatment-related complications, or withdrawals attributable to the intervention were observed. All participants tolerated the intervention well, and no participant discontinued the study because of discomfort associated with the device.

### Influence of the ITSS intervention on subjectively felt nasal congestion and sleep state

According to Table 2, no significant difference was observed between the two groups in the perceived level of nasal congestion prior to the intervention. Following the intervention, the CON exhibited an increase in perceived nasal congestion, whereas the ITSS group showed a decrease, yielding significant differences between groups, within groups, and in the interaction. The perceived sleep condition upon awakening showed no significant difference between the two groups before the intervention. Following the intervention, however, the CON demonstrated an increase in perceived sleep condition, while the ITSS group reported a decrease, leading to significant effects between groups, within groups, and in the interaction effect.

**Table 2:** Comparative results of subjective nasal congestion and sleep condition

		Groups (G)						
		Control		ITSS			*p* *(η²)*	
Items	Time (T)	mean	SD	mean	SD	G	*T*	G×T
Nasal congestion	Pre	4.81	0.98	4.93	0.97	0.001	0.004	0.001
	Post	5.22	0.73	3.55	1.21	(0.213)	(0.133)	(0.344)
Sleep condition	Pre	4.64	1.79	4.77	1.53	0.016	0.006	0.001
	Post	4.93	0.89	3.36	0.99	(0.096)	(0.123)	(0.243)

All data are presented as mean and standard deviation (SD). *CON* control, *ITSS* intranasal thermal steam spray

**Fig. 2:**
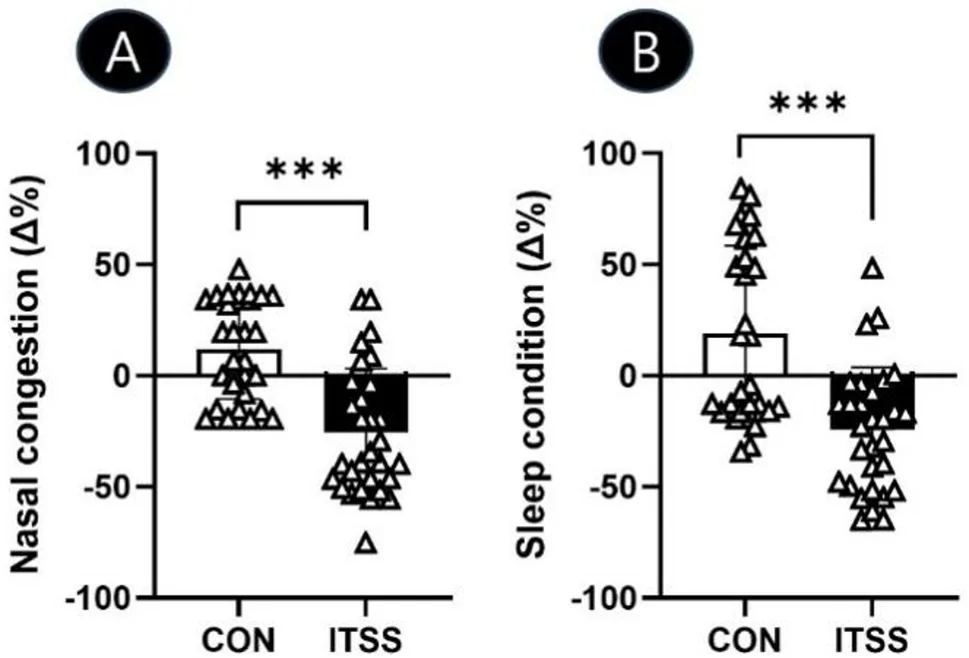
Differences of Δ% in perceived nasal congestion and sleep condition. CON, control group; ITSS, intranasal thermal steam spray group. ^***^ indicates *p* < 0.001 between groups

As illustrated in Fig. 2, changes in perceived nasal congestion and sleep condition following the intervention differed significantly between the groups. In the CON, nasal congestion increased by 11.92 ± 22.28%, whereas it decreased by -25.55 ± 28.74% in the ITSS group, showing a significant difference between the groups (Fig. 2A). Similarly, subjective sleep condition increased by 18.88 ± 39.59% in the CON but declined by -24.22 ± 28.03% in the ITSS group, resulting in a statistically significant difference (Fig. 2B).

### Influence of the ITSS intervention on respiratory capacity and BP

As observed in Table 3, there was no significant difference in resting respiratory rate between the two groups prior to the intervention. However, after the intervention, the resting respiratory rate increased in the CON but decreased in the ITSS group, resulting in a significant interaction effect. Similarly, no significant differences were observed between the groups in FEV₁ and PEF before the intervention. Following the intervention, however, both FEV₁ and PEF decreased in the CON but increased in the ITSS group, yielding statistically significant interaction effects. As further observed in Table 3, there were no significant differences in systolic blood pressure (SBP) and diastolic blood pressure (DBP) between the groups before the intervention. However, after the intervention, SBP and DBP increased in the CON but decreased in the ITSS group, resulting in significant interaction effects.

**Table 3:** Comparative results of respiratory capacity and blood pressure

		Groups (G)						
		CON		ITSS		*p* *(η²)*		
Items	Time (T)	mean	SD	mean	SD	G	*T*	G×T
*Respiratory capacity*								
rRR (reps/min)	Pre	17.13	3.18	17.70	3.27	0.055	0.793	0.001
	Post	18.90	2.43	15.67	4.05	(0.062)	(0.001)	(0.196)
FEV₁ (L)	Pre	3.20	0.68	3.26	0.75	0.031	0.212	0.001
	Post	2.95	0.66	3.66	0.76	(0.078)	(0.027)	(0.320)
PEF (L/s)	Pre	4.08	1.24	4.05	0.73	0.061	0.002	0.001
	Post	3.89	1.27	4.97	1.26	(0.059)	(0.159)	(0.298)
*Blood pressure level*								
SBP (mmHg)	Pre	131.47	11.04	132.77	10.03	0.053	0.835	0.001
	Post	137.30	12.76	126.40	8.16	(0.063)	(0.001)	(0.283)
DBP (mmHg)	Pre	74.57	9.29	76.20	10.89	0.771	0.315	0.036
	Post	75.77	9.03	72.87	7.63	(0.001)	(0.017)	(0.074)

All data are presented as mean and standard deviation (SD). *CON* control, *ITSS* intranasal thermal steam spray, *rRR* resting respiratory rate, *FEV₁* forced expiratory volume in 1s, *PEF* peak expiratory flow, *SBP* systolic blood pressure, *DBP* diastolic blood pressure

**Fig. 3:**
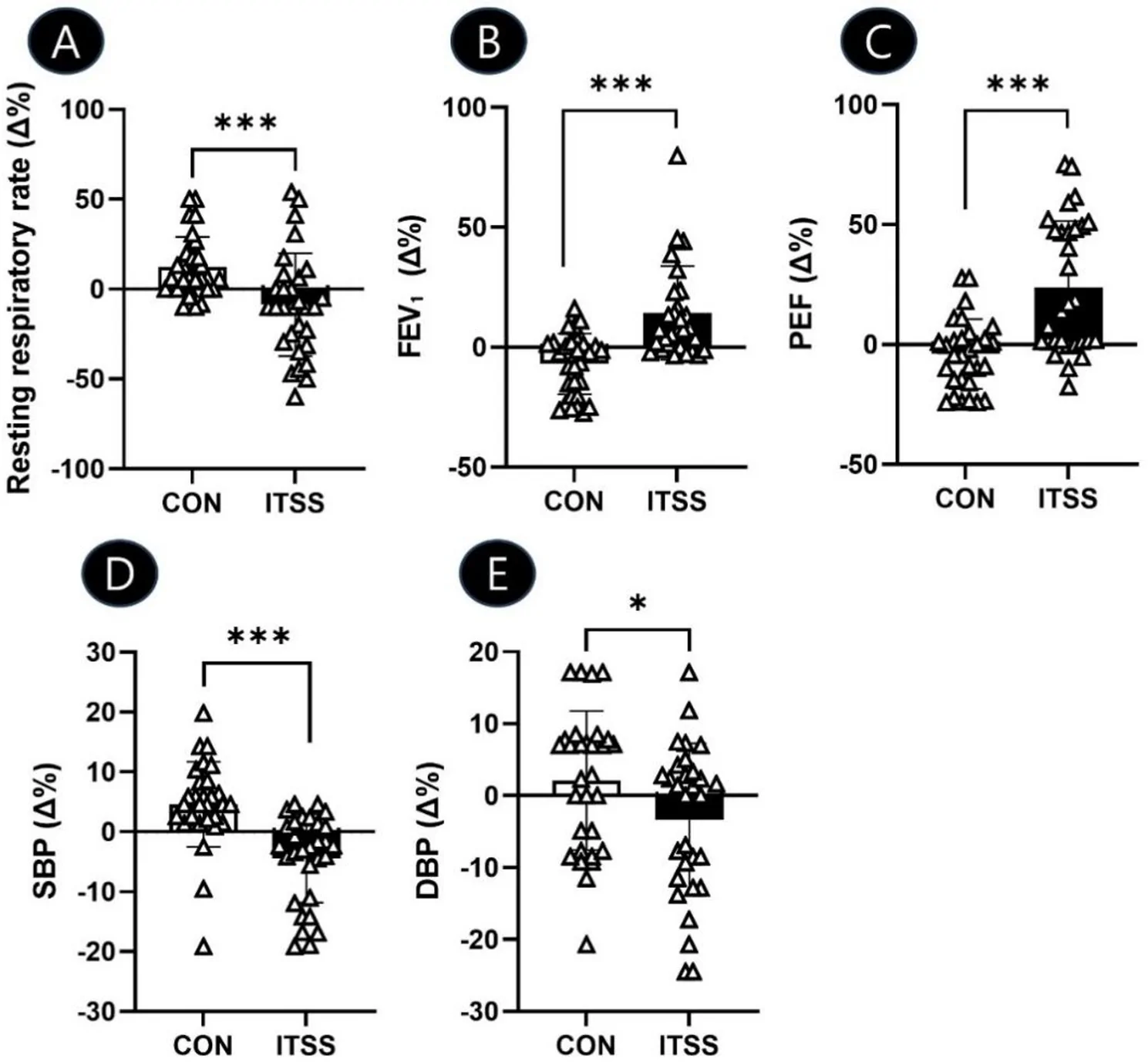
Differences of Δ% in respiration capacity and BP levels. CON, control group; ITSS, intranasal thermal steam spray group; FEV₁, forced expiratory volume in 1 s; PEF, peak expiratory flow; SBP, systolic blood pressure; DBP, diastolic blood pressure. ^*^ and ^***^ indicate *p* < 0.05 and *p* < 0.001 between groups

As illustrated in Fig. 3, the changes in respiratory capacity and BP levels after the intervention revealed significant differences between the groups. The resting respiratory rate increased by 12.39 ± 16.70% in the CON, while it decreased by -8.64 ± 28.56% in the ITSS group, indicating a significant between-group difference (Fig. 3A). Regarding pulmonary function, FEV₁ decreased by -6.98 ± 12.69% in the CON but increased by 14.29 ± 19.54% in the ITSS group (Fig. 3B). Similarly, PEF declined by -3.99 ± 14.55% in the CON but rose by 23.80 ± 27.60% in the ITSS group (Fig. 3C), both showing significant differences between the groups. As for BP, SBP increased by 4.58 ± 7.10% in the CON, whereas it decreased by -4.44 ± 7.38% in the ITSS group (Fig. 3D). DBP increased by 2.11 ± 9.66% in the CON and by -3.35 ± 10.60% in the ITSS group (Fig. 3E), both also showing significant differences between the groups. These findings suggest that the application of ITSS may have a beneficial effect on cardiopulmonary function.

### Influence of the ITSS intervention on HRV

As shown in Table 4, the HR, a time-domain index of HRV, did not differ significantly between the two groups before the intervention. However, after the intervention, HR increased in the CON but decreased in the ITSS group, resulting in a significant interaction effect. SDNN also showed no significant difference between the two groups prior to the intervention. After the intervention, both groups exhibited a decreasing trend, but the CON demonstrated a more pronounced decline compared to the ITSS group, leading to significant differences within groups and in the interaction effect. Meanwhile, RMSSD did not significantly differ between the groups before the intervention. After the intervention, however, RMSSD decreased in the CON and increased in the ITSS group, showing significant differences between groups and in the interaction effect. As further shown in Table 4, the frequency-domain indices of HRV, namely LF and the LF/HF ratio, did not show significant differences between the two groups before the intervention. However, after the intervention, both LF and the LF/HF ratio increased in the CON but decreased in the ITSS group, resulting in significant between-group and interaction effects. Meanwhile, HF also showed no significant difference between the groups prior to the intervention. After the intervention, however, HF decreased in the CON and increased in the ITSS group, yielding statistically significant differences between groups, within groups, and in the interaction effect.

**Table 4:** Comparative results of HRV

		Groups (G)						
		CON		ITSS		*p* *(η²)*		
Items	Time (T)	mean	SD	mean	SD	G	*T*	G×T
*Time domain of heart rate variability*								
HR (bpm)	Pre	76.57	11.33	75.33	14.88	0.071	0.143	0.001
	Post	79.00	10.25	69.83	9.44	(0.055)	(0.037)	(0.203)
SDNN (ms)	Pre	73.37	37.57	69.22	31.30	0.351	0.001	0.041
	Post	41.69	14.05	56.63	24.76	(0.015)	(0.291)	(0.071)
RMSSD (ms)	Pre	35.53	10.98	36.53	11.99	0.013	0.665	0.001
	Post	30.18	11.71	43.01	11.45	(0.103)	(0.003)	(0.267)
*Frequency domain of heart rate variability*								
LF (ms²)	Pre	657.17	98.57	663.17	109.65	0.031	0.315	0.001
	Post	697.70	116.14	592.37	90.57	(0.078)	(0.017)	(0.194)
HF (ms²)	Pre	711.97	155.93	713.33	127.96	0.001	0.001	0.001
	Post	543.37	163.60	771.07	104.90	(0.174)	(0.187)	(0.489)
LF/HF ratio	Pre	2.40	0.83	2.36	1.05	0.001	0.001	0.001
	Post	2.70	0.79	0.78	0.17	(0.363)	(0.372)	(0.563)

All data are presented as mean and standard deviation (SD). *CON* control, *ITSS* intranasal thermal steam spray, *SBP* systolic blood pressure, *DBP* diastolic blood pressure, *HR* heart rate, *SDNN* standard deviation of NN intervals, *RMSSD* root mean square of successive differences, *LF* low frequency, *HF* high frequency, *ANS* autonomic nervous system

**Fig. 4:**
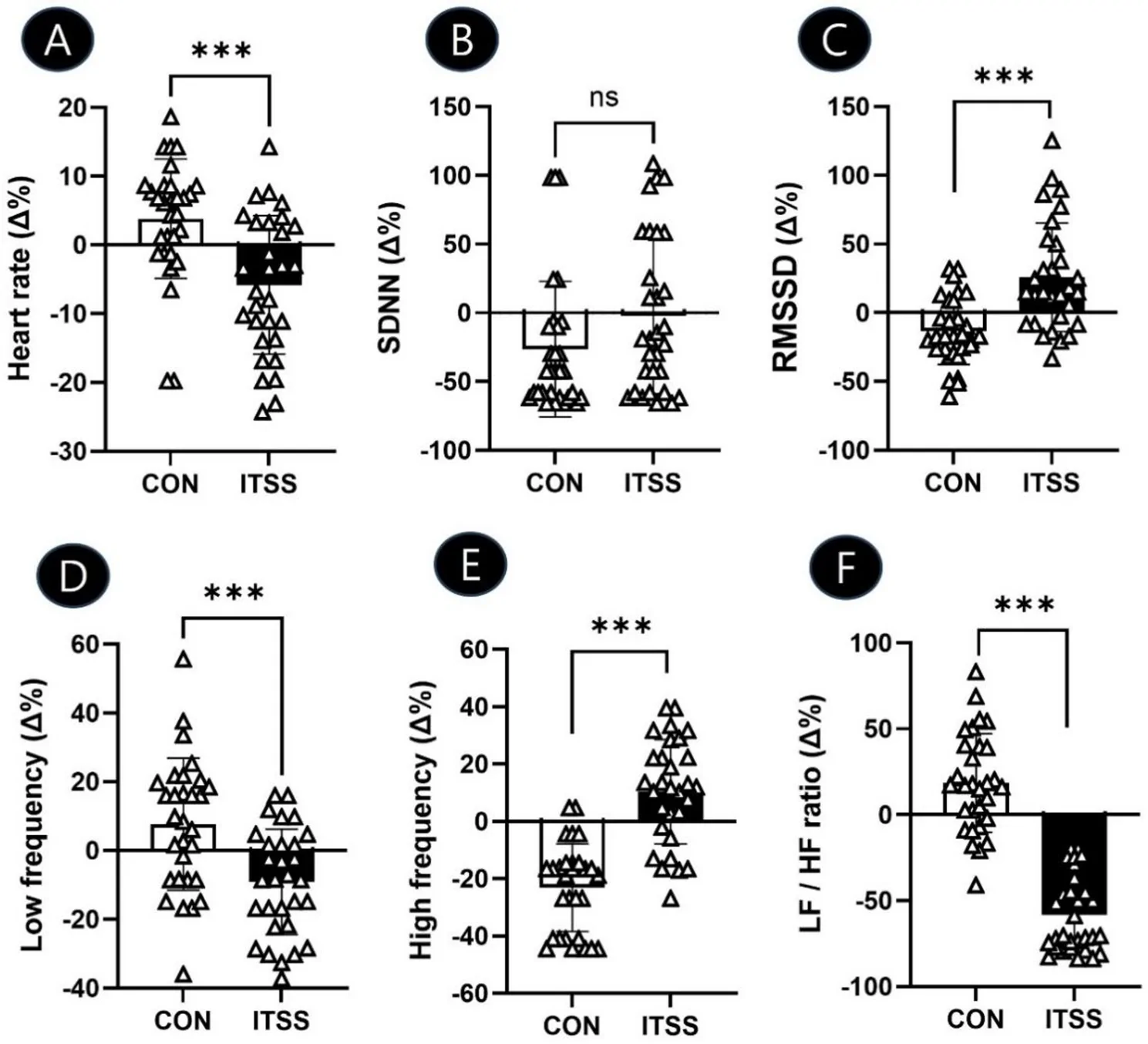
Differences of Δ% in heart rate variability of time and frequency domains. CON, control group; ITSS, intranasal thermal steam spray group; SDNN, standard deviation of NN intervals; RMSSD, root mean square of successive differences; LF, low frequency; HF, high frequency. ^ns^ and ^***^ indicate *p* > 0.05 and *p* < 0.001 between groups

As illustrated in Fig. 4, HR increased by 3.82 ± 8.67% in the CON but decreased by -5.83 ± 10.09% in the ITSS group, resulting in a significant difference (Fig. 4A). Although SDNN decreased by -26.29 ± 49.35% in the CON and by -2.17 ± 56.74% in the ITSS group, this difference was not statistically significant (Fig. 4B). In contrast, RMSSD declined by -13.38 ± 24.29% in the CON but increased by 25.75 ± 39.49% in the ITSS group, showing a significant difference between the groups (Fig. 4C). LF power increased by 7.67 ± 19.22% in the CON and decreased by -9.15 ± 15.35% in the ITSS group, yielding a significant difference (Fig. 4D). HF power decreased by -23.12 ± 15.30% in the CON but increased by 10.37 ± 18.19% in the ITSS group, also showing a significant difference (Fig. 4E). Lastly, the LF/HF ratio rose by 18.47 ± 28.77% in the CON but dropped by -58.17 ± 22.02% in the ITSS group, resulting in a statistically significant difference (Fig. 4F).

### Influence of the ITSS intervention on EEG factors

As presented in Table 5, following the intervention, the wide band mean increased in the CON but decreased in the ITSS group, resulting in a significant interaction effect. Although no statistically significant post-intervention differences were observed in delta, theta, beta, or other wave ratios, alpha power decreased in the CON but increased in the ITSS group, showing a significant interaction effect (*p* = 0.023). Similarly, Gamma power showed a greater increase in the ITSS group compared to the CON after the intervention, indicating a significant interaction effect (*p* = 0.038).

**Table 5:** Comparative results of EEG factors

		Groups (G)						
		CON		ITSS		*p* *(η²)*		
Waves/Power	Time (T)	mean	SD	mean	SD	G	*T*	G×T
Wide band (µV²)	Pre	29.16	12.08	30.33	9.29	0.112	0.852	0.001
	Post	33.80	9.76	25.20	9.72	(0.043)	(0.001)	(0.192)
Delta	Pre	14.30	4.12	14.23	3.51	0.071	0.025	0.224
	Post	10.61	3.85	13.11	6.90	(0.056)	(0.084)	(0.025)
Theta	Pre	14.85	5.81	13.86	6.27	0.466	0.957	0.106
	Post	12.97	5.76	15.87	8.85	(0.009)	(0.001)	(0.045)
Alpha	Pre	11.38	4.15	10.46	4.21	0.835	0.673	0.023
	Post	10.05	3.40	11.38	5.01	(0.001)	(0.003)	(0.086)
Beta	Pre	9.01	3.78	8.26	3.85	0.017	0.023	0.089
	Post	12.05	4.86	8.72	4.74	(0.094)	(0.086)	(0.049)
Gamma	Pre	1.69	0.35	1.74	0.39	0.066	0.139	0.038
	Post	1.65	0.30	1.98	0.71	(0.057)	(0.037)	(0.072)
Alpha/Beta ratio	Pre	1.01	0.46	1.06	0.42	0.705	0.634	0.364
	Post	1.13	0.47	1.02	0.50	(0.002)	(0.004)	(0.014)
Theta/Beta ratio	Pre	1.66	0.36	1.65	0.31	0.209	0.612	0.301
	Post	1.79	0.56	1.61	0.51	(0.027)	(0.004)	(0.018)

All data are presented as mean and standard deviation (SD). *CON* control, *ITSS* intranasal thermal steam spray

**Fig. 5:**
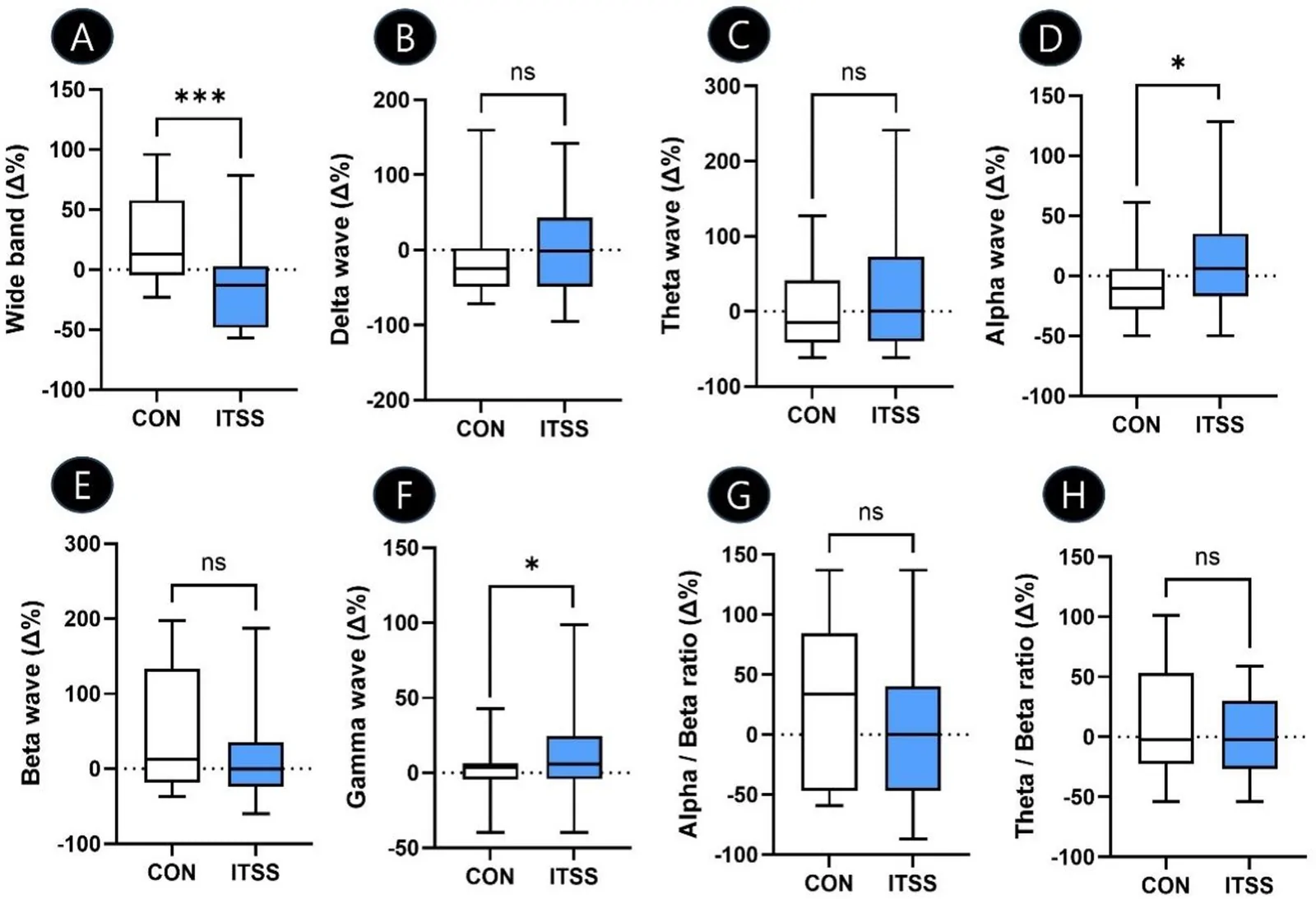
Differences of Δ% in electroencephalogram factors. CON, control group; ITSS, intranasal thermal steam spray group. ^ns, *,^ and ^***^ indicate *p* > 0.05, *p* < 0.05, and *p* < 0.001 between groups

As shown in Fig. 5, key changes in EEG components after the intervention demonstrated notable group differences. The wide band power increased by 24.90 ± 38.15% in the CON but decreased by -13.46 ± 33.19% in the ITSS group (Fig. 5A). Alpha power declined by about − 3.81 ± 32.46% in the CON but increased by 14.90 ± 43.56% in the ITSS group (Fig. 5D), and gamma power showed a slight increase of 0.20 ± 19.66% in the CON compared to a 13.71 ± 32.89% increase in the ITSS group (Fig. 5F), all of which resulted in significant differences between the groups. Meanwhile, delta (Fig. 5B), theta (Fig. 5C), and beta powers (Fig. 5E), as well as the ABR (Fig. 5G) and TBR (Fig. 5H), showed changes within each group after the intervention, but no significant differences were observed between the groups.

### Influence of the ITSS intervention on sleep factors

As presented in Table 6, the sleep quality component of the PSQI-K showed no significant difference between the two groups prior to the intervention. After the intervention, sleep quality increased in the CON but decreased in the ITSS group, indicating a significant interaction effect. Similar trends were observed in sleep-latency, duration, efficiency, disturbance, and daytime dysfunction, suggesting an overall improvement in sleep quality in the ITSS group. Accordingly, the sum scores of the PSQI-K significantly improved in the ITSS group compared to the CON.

**Table 6:** Comparative results of PSQI-K factors between groups

		Groups (G)						
		CON		ITSS		*p* *(η²)*		
Items	Time (T)	mean	SD	mean	SD	G	*T*	G×T
Sleep quality	Pre	2.07	0.58	2.10	0.55	0.001	0.015	0.001
	Post	2.73	0.45	0.97	0.61	(0.526)	(0.098)	(0.617)
Sleep latency	Pre	3.03	1.13	3.10	1.37	0.005	0.183	0.001
	Post	3.60	1.30	2.00	1.26	(0.129)	(0.031)	(0.234)
Sleep duration	Pre	1.70	0.60	1.67	0.66	0.028	0.556	0.001
	Post	1.97	0.67	1.30	0.79	(0.081)	(0.006)	(0.195)
Sleep efficiency	Pre	2.10	0.84	2.13	0.68	0.001	0.001	0.001
	Post	2.36	0.73	0.96	0.93	(0.246)	(0.168)	(0.333)
Sleep disturbance	Pre	2.17	0.70	2.23	0.57	0.001	0.002	0.001
	Post	2.37	0.85	1.27	0.78	(0.176)	(0.150)	(0.291)
Sleep medication	Pre	0.13	0.43	0.17	0.53	1.000	0.155	0.155
	Post	0.13	0.43	0.10	0.31	0.000	(0.034)	(0.034)
Daytime dysfunction	Pre	2.37	0.81	2.40	0.72	0.001	0.006	0.001
	Post	2.57	0.57	1.40	0.67	(0.301)	(0.122)	(0.239)
Sum scores of PSQI-K	Pre	11.50	1.46	11.70	2.26	0.001	0.001	0.001
	Post	13.00	2.04	7.03	2.31	(0.453)	(0.292)	(0.608)

All data are presented as mean and standard deviation (SD). *CON* control, *ITSS* intranasal thermal steam spray, *PSQI* Pittsburgh Sleep Quality Index, *VAS* visual analogue scale

**Fig. 6:**
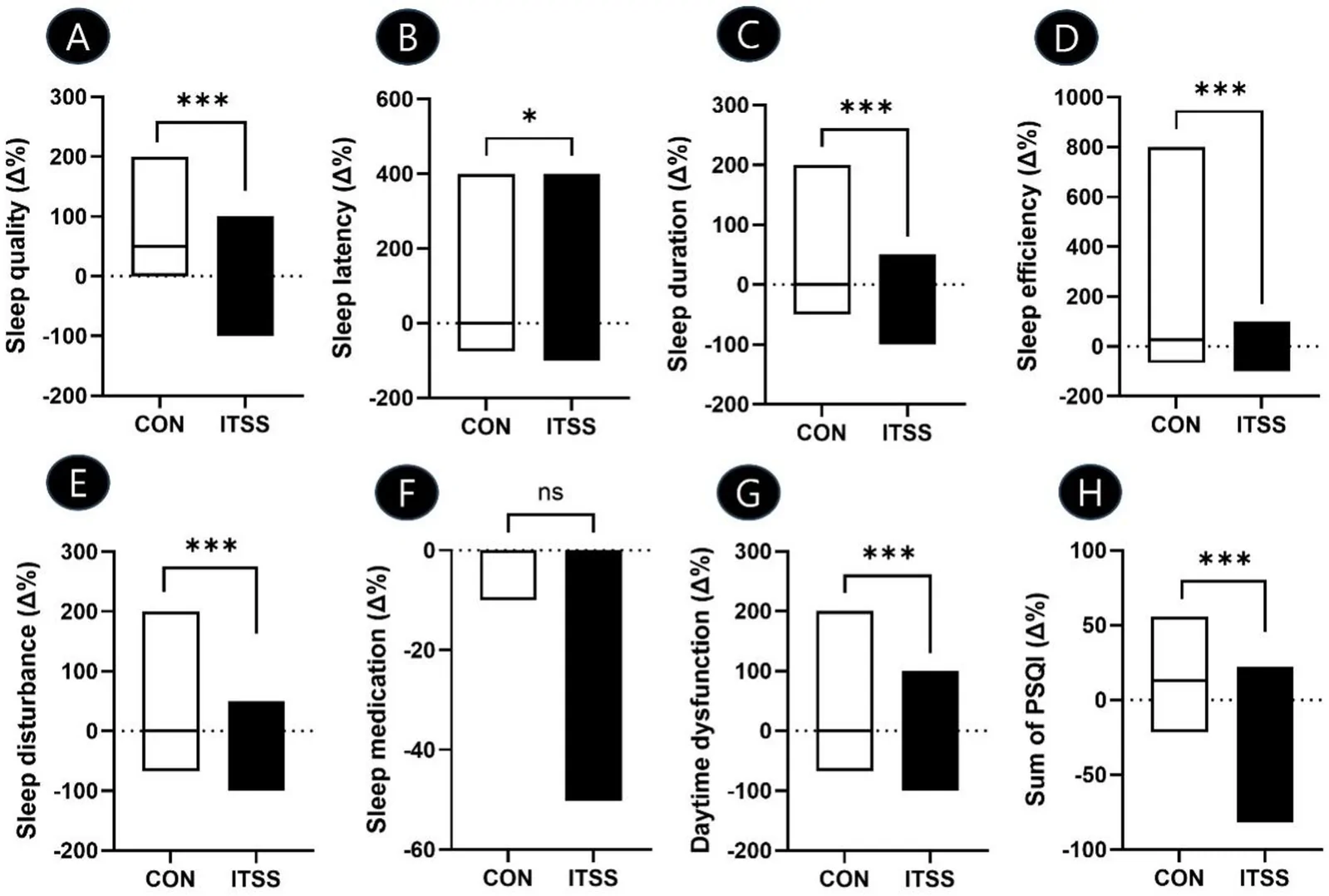
Differences of Δ% in PSQI-K. CON, control group; ITSS, intranasal thermal steam spray group. ^*^ and ^***^ indicate *p* < 0.05 and *p* < 0.001 between groups

As shown in Fig. 6, sleep quality increased by 41.67 ± 43.71% in the CON, whereas it decreased by -48.33 ± 47.82% in the ITSS group (Fig. 6A). Sleep latency also increased by 35.61 ± 84.19% in the CON but decreased by -21.78 ± 86.0% in the ITSS group, showing a significant difference (Fig. 6B). Sleep duration (Fig. 6C), sleep efficiency (Fig. 6D), and sleep disturbance (Fig. 6E) increased by 26.11 ± 55.48%, 56.11 ± 155.52%, and 24.44 ± 72.42%, respectively, in the CON, while decreasing by -25.0 ± 40.29%, -50.89 ± 55.16%, and − 44.45 ± 34.0% in the ITSS group, all indicating significant differences between the two groups. No notable changes were observed in the use of sleep medication (Fig. 6F). However, daytime dysfunction increased by 35.0 ± 87.68% in the CON and decreased by -32.78 ± 41.40% in the ITSS group, revealing a statistically significant difference (Fig. 6G). Finally, the total PSQI-K score increased by 14.64 ± 22.44% in the CON but decreased by -38.90 ± 21.99% in the ITSS group, resulting in a significant difference (Fig. 6H).

## Discussion

The principal finding of the present study was that improving the nasal environment through ITSS significantly enhanced subjective and objective indicators of sleep quality. Improvements in respiratory function, ANS regulation, and brain activity appear to represent potential physiological mechanisms underlying these beneficial effects on sleep. Notably, improved nasal environment was associated with stabilization of the ANS and enhanced relaxation, as evidenced by changes in wide band, alpha, and gamma EEG activity in young adults with nasal congestion and irregular sleep patterns. These neurophysiological responses appear to contribute to the observed improvements in sleep quality.

The nasal environment, including factors such as nasal cavity volume and resistance, plays a significant role in the transition from wakefulness to sleep. Just as individuals maintain various postures—such as sitting, reclining, or standing—while awake, they also adopt different positions during sleep. These postural changes, along with stressors or neurological factors that may occur during the day, can influence nasal cavity volume and, consequently, affect sleep quality. In this context, an epidemiological study by Young et al. [38] identified nasal congestion as a risk factor for sleep-disordered breathing. In a subsequent population-based cohort study with a five-year follow-up [39], chronic nocturnal nasal congestion was found to be a significant risk factor for habitual snoring (odds ratio 4.9). Beyond epidemiological findings demonstrating the association between nasal congestion and sleep disorders, several laboratory studies have provided experimental evidence for this relationship. Eckert and Younes [40] experimentally induced nasal obstruction and demonstrated an increase in both sleep apnea and electroencephalographic arousals during sleep. Similarly, Fitzpatrick et al. [41] showed that modulating nasal resistance using irritants led to the induction of apnea and hypopnea during sleep. Collectively, the existing body of research suggests that nasal congestion—regardless of its underlying cause—can be considered a contributing factor to the development of sleep problems. Even within the present study, among the 60 participants, the average duration of nasal congestion was 3.39 ± 0.74 years, while the duration of sleep irregularity associated with sleep disturbances was 3.01 ± 0.77 years. These findings suggest a potential relationship between nasal congestion—such as that caused by rhinitis—and sleep-related problems. Considering that the participants were in their early to mid-20s, this association may indicate the potential for more serious consequences later in life. Although not addressed as a primary outcome in this study, the correlation between nasal congestion and irregular sleep patterns was examined, revealing a statistically significant association (*r* = 0.308, *p* = 0.017), suggesting a clear link between nasal congestion and sleep patterns. Additionally, prior to the intervention, the average severity of nasal congestion assessed by VAS among the sixty participants was 4.87 ± 0.97, while the perceived severity of sleep-related problems was 4.71 ± 1.64. These findings further support the notion that nasal congestion is closely associated with subjective sleep difficulties. Although no model has directly explained or demonstrated that changes in nasal resistance can induce sleep problems, considering the Starling resistor model in relation to nasal resistance provides a possible explanation. If a narrowed region within the nasal cavity or any other cause leads to increased nasal resistance, negative pressure may develop downstream of the affected area. This negative pressure could contribute to the collapse of the oropharyngeal region, which lacks skeletal support [42]. Furthermore, during sleep, the muscles comprising the oropharyngeal region are in a more relaxed state compared to wakefulness, making them more susceptible to collapse due to negative pressure [43].

To date, numerous studies have reported that treating nasal congestion can improve the quality of life in patients with sleep problems. Li et al. [44] reported an improvement in patients’ quality of life following nasal surgery. Similarly, Miyazaki et al. [45] conducted a study on 54 obstructive sleep apnea patients using a catheter-based pressure transducer to examine the relationship between nasal congestion and obstructive sleep apnea. Their findings suggested that while nasal congestion itself may not be a primary cause of obstructive sleep apnea, it can act as a contributing factor. Additionally, Löth et al. [46] demonstrated that the use of a nostril dilator increased nasal breathing in snoring patients, leading to improved quality of life. Recently, the ITSS device, developed to alleviate nasal congestion, has been reported to help relieve congestion by delivering warm water to the nasal cavity using jet stream wet technology [17]. However, the underlying mechanisms through which ITSS influences sleep quality remain largely unexplored. Specifically, how ITSS improves respiratory function by enhancing nasal hydration and the perception of nasal openness—and how these changes subsequently affect the ANS, brain activity, and ultimately sleep patterns—has not yet been fully investigated. To address this, the present study investigated the effects of ITSS treatment over an eight-week period. The results showed that nasal congestion severity, as assessed by the VAS, increased by approximately 12% in the CON, whereas the ITSS group experienced a 26% reduction following ITSS treatment. Similarly, sleep condition worsened by around 19% in the CON but improved by about 24% in the ITSS group. These improvements were also reflected in respiratory function: the resting respiratory rate increased by approximately 12% in the CON, whereas it decreased by nearly 9% in the ITSS group. Furthermore, while FEV₁ and PEF showed minimal change or slight decreases in the CON, the ITSS group demonstrated significant increases of about 14% and 24%, respectively, suggesting that the improvement in nasal congestion contributed to enhanced respiratory function. This improvement in respiratory function appeared to contribute to reductions in BP, as evidenced by ANS markers in the ITSS group. Specifically, a decrease in SDNN and an increase in RMSSD were observed, indicating a shift toward parasympathetic nervous dominance. Moreover, HF power, which reflects parasympathetic nervous activity, decreased by approximately 23% in the CON but increased by around 10% in the ITSS group. In contrast, the LF/HF ratio, representing sympathetic nervous activity, increased by about 18% in the CON but decreased by nearly 58% in the ITSS group. These results suggest that the ITSS intervention may have facilitated ANS stabilization and contributed to improved BP regulation. The observed improvements in respiratory function may have contributed to enhanced autonomic regulation by reducing upper airway resistance and improving breathing efficiency [47]. This interpretation is supported by the concurrent improvements in heart rate variability and blood pressure observed in the present study, suggesting a shift toward greater parasympathetic activity and reduced sympathetic dominance. Because autonomic balance plays an important role in sleep regulation [9], these physiological changes may partially explain the improvement in sleep quality following intranasal thermal steam spray.

To date, no research has directly examined the effects of ITSS on nasal congestion relief and brain wave changes during sleep or daytime activities such as working. However, a substantial body of literature has investigated the relationship between nasal congestion, sleep quality, and brain wave activity. Understanding these connections is crucial, as nasal congestion can significantly impact sleep architecture, cognitive function, and overall well-being. Several studies have explored the effects of improving nasal environment on brain wave characteristics and sleep patterns. Walsh et al. [48] reported that nasal expansion led to an increase in delta wave activity, which is closely associated with deep sleep and physical recovery. Delta waves (0.5–4 Hz) play a fundamental role in restorative sleep, facilitating tissue repair, immune function, and overall physiological recovery [49–51]. The enhancement of delta wave activity suggests that nasal expansion could contribute to improved sleep quality and physical restoration. According to the EEG analysis in this study, the delta wave increased by nearly 3.23% in the ITSS group, whereas it significantly decreased by approximately 11% in the sham CON (*p* = 0.025). This trend appears to be consistent with previous studies; however, no significant interaction effect was observed. Therefore, further in-depth investigations are warranted to elucidate the specific mechanisms underlying these changes. This suggests that intranasal steam spray may help regulate sleep-related brain activity by increasing body temperature and alleviating nasal congestion to some extent. This trend is further supported by the findings in the ITSS group, where alpha wave, related to relaxation and a calm mental state, improved by around 15% after the ITSS intervention (interaction effect *p* = 0.023). Moreover, gamma wave, which is closely linked to stress, anxiety, and cognitive processing, increased by around 14% (interaction effect *p* = 0.038). Meanwhile, wide band mean power typically ranges between 20 and 40 µV² in a normal resting state. In this study, the CON showed a 25% increase from 29.16 ± 12.08 µV² at baseline to 33.80 ± 9.76 µV² post-intervention, indicating a shift toward a heightened arousal state. In contrast, the ITSS group exhibited a 13% decrease from 30.33 ± 9.29 µV² to 25.20 ± 9.72 µV², suggesting a transition toward a more relaxed physiological state. These results suggest that ITSS may help reduce psychological stress and alleviate mental tension by moistening and expanding the nasal passages. In relation to this, several previous studies have indicated that theta wave plays a crucial role in the transition between wakefulness and sleep, supporting memory processing and emotional regulation [52]. In other words, an increase in theta wave activity suggests that nasal expansion may facilitate smoother sleep transitions and enhance cognitive processing during sleep. Additionally, alpha wave, which is associated with relaxation, meditation, and the transition to sleep, has been shown to increase after nasal expansion. Since alpha wave activity is often linked to a state of wakeful rest and is considered essential for initiating sleep [53, 54], the observed increase in alpha wave in this study indicates that nasal expansion promotes psychological relaxation and prepares the brain for sleep onset. Furthermore, the findings align with the study by Hammer et al. [55], which reported that gamma wave—frequently activated during REM sleep and associated with high-order cognitive functions such as emotional regulation and problem-solving—increased with nasal expansion. In this study, beta wave activity showed a notable increase in the CON, whereas a relatively smaller increase was observed in the ITSS group. This pattern may suggest that the ITSS group experienced a more stable and calmer psychological state. Supporting this, Pei et al. [52] reported that reduced beta wave, resulting from nasal expansion, contributes to a more relaxed physiological state, potentially mitigating hyperarousal that can hinder sleep onset and maintenance. Similarly, some of research noted that beta activity plays a critical role in motor and sensory regulation, as well as in stabilizing and maintaining sleep by ensuring efficient neural communication during sleep [54]. Ultimately, improved nasal condition induced by ITSS may positively influence beta wave, which is associated with physical stability and improved concentration. However, as both groups in the present study showed increased beta waves with no statistically significant interaction effect, further research is warranted to better understand the underlying mechanisms.

To our knowledge, no studies have directly examined the effects of ITSS on nasal congestion relief and PSQI. However, it is well known that nasal congestion negatively affects sleep quality, and various nasal sprays have been reported to help alleviate this issue. In other words, nasal congestion can cause breathing difficulties during sleep, leading to a decline in sleep condition and quality, which may be reflected in an increased PSQI score. For example, saline sprays are commonly used to rinse the nasal passages, remove allergens, and alleviate dryness inside the nasal cavity [56, 57]. Hermelingmeier et al. [58] highlighted that intranasal saline therapy is a safe, cost-effective, and efficient treatment, as it helps remove mucus, accumulated secretions, and inflammatory cytokines from the nasal passages. Similarly, Fokkens et al. [59] reported that it supports mucociliary clearance and reduces mucosal swelling. As a result, intranasal saline therapy is widely recommended as an adjunct treatment for sinonasal conditions such as rhinosinusitis, rhinitis, and upper respiratory tract infections. The intranasal steam spray used in this study introduces 43 °C water into the nasal cavity, promoting nasal expansion and increasing cranial temperature, which may help induce sleep. Notably, the results of this study showed that the “sleep quality” score on the PSQI-K improved by approximately 48% following ITSS intervention, whereas the sham CON exhibited a deterioration of about 42%. Moreover, in terms of sleep latency—the time taken to fall asleep—the CON showed an increase of approximately 36% after the intervention, whereas the ITSS group exhibited a decreasing trend of about 22%, approaching the critical threshold of falling asleep within 30 min. In addition, regarding the PSQI-K subscales—“sleep duration,” “sleep efficiency”, “sleep disturbances,” and “daytime dysfunction”—the placebo-controlled group showed an increasing trend after the intervention, whereas the ITSS group exhibited significant reductions, indicating an interaction effect. Consequently, the total PSQI-K score related to sleep quality increased by approximately 15% in the CON but decreased by 39% in the ITSS group, approaching the threshold score of 7. Considering the PSQI-K criteria established by Sohn et al. [36], where a score of 8.5 or lower indicates good sleep quality and a score above 8.5 indicates poor sleep quality, these findings strongly suggest that intranasal thermal steam spray has a highly positive impact on sleep quality. Meanwhile, a study using the NOZOVENT nasal dilator in 15 individuals with non-pathological nasal structures reported no significant changes in apnea–hypopnea events or oxygen saturation, with only a limited reduction in snoring episodes during slow-wave sleep [60]. Consistently, a systematic review found that most studies reported no improvement in AHI with external nasal dilators, while minor improvements were observed only with certain internal dilators [61]. Taken together, these findings suggest that the effects of nasal dilators on objective sleep parameters remain inconclusive, underscoring the need for further well-designed studies to establish their clinical significance. Several limitations of the present study should be acknowledged. First, although the study was prospectively designed with an a priori sample size calculation and enrolled the required number of participants according to the study design, the overall sample size remained relatively modest. Moreover, the sample size was estimated for the overall two-group, two-time-point repeated-measures design rather than for an outcome-specific effect estimate. Consequently, the study may not have been adequately powered to detect differences across all secondary and mechanistic outcomes, and these findings should therefore be interpreted as supportive and exploratory. Future trials should prospectively define a single primary outcome and base the sample size calculation on a clinically meaningful expected difference in that outcome. Second, because the participants were limited to young adults in their twenties with nasal congestion and irregular sleep patterns, the generalizability of the findings to broader populations, including older adults and patients with clinically diagnosed nasal or sleep disorders, is limited. Accordingly, future studies should recruit larger and more diverse populations to improve the external validity of the findings. Third, although each outcome measure represented a prespecified physiological or clinical endpoint, multiple statistical analyses were performed across several outcome domains. Therefore, the possibility of type I error inflation cannot be entirely excluded and should be considered when interpreting the results. Finally, the intervention was well tolerated throughout the 8-week study period, with no intervention-related serious adverse events or withdrawals attributable to the use of the temperature-controlled intranasal thermal steam spray. While these findings provide preliminary evidence supporting the short-term safety of the intervention, larger-scale clinical trials with longer follow-up periods are warranted to further establish both its long-term safety and clinical effectiveness.

## Conclusions

This study concluded that the application of ITSS enhanced the nasal environment—particularly by alleviating the sensation of nasal congestion—which in turn led to favourable changes in cardiopulmonary function. These physiological improvements may further contribute to better sleep quality by promoting ANS balance and stabilizing brain activity.

## Supplementary Information

Supplementary Material 1.

Supplementary Material 2.

Supplementary Material 3.

## Data Availability

The data supporting the findings of this study are available within the article and its supplementary materials.

## Abbreviations

ANS: Autonomic nervous system
ABR: Alpha/Beta ratio
BMI: Body mass index
BP: Blood pressure
CON: Control group
CRIS: Clinical Research Information Service
DBP: Diastolic blood pressure
EEG: Electroencephalography
FEV₁: Forced expiratory volume in one second
FFT: Fast Fourier Transform
HF: High frequency
HR: Heart rate
HRV: Heart rate variability
ITSS: Intranasal thermal steam spray
LF: Low frequency
PA: Physical activity
PEF: Peak expiratory flow
PSQI: Pittsburgh Sleep Quality Index
PSQI-K: Korean version of the Pittsburgh Sleep Quality Index
rRR: Resting respiratory rate
RMSSD: Root mean square of successive differences
SD: Standard deviation
SDNN: Standard deviation of normal-to-normal intervals
TBR: Theta/Beta ratio
VAS: Visual analogue scale
WHO: World Health Organization
